# The Effects of Temporal and Spatial Predictions on Stretch Reflexes of Ankle Flexor and Extensor Muscles While Standing

**DOI:** 10.1371/journal.pone.0158721

**Published:** 2016-07-06

**Authors:** Kimiya Fujio, Hiroki Obata, Noritaka Kawashima, Kimitaka Nakazawa

**Affiliations:** 1 Sports Science Laboratory, Department of Life Sciences, Graduate School of Arts and Sciences, University of Tokyo, Tokyo, Japan; 2 Department of Rehabilitation for the Movement Functions, Research Institute of the National Rehabilitation Center for Persons with Disabilities, Tokorozawa, Japan; Duke University, UNITED STATES

## Abstract

The purpose of the present study was to investigate how stretch reflex (SR) responses in the ankle extensor (soleus: SOL) and flexor (tibialis anterior: TA) muscles would be modulated with temporal and/or spatial predictions of external perturbations and whether their effects are specific to the standing posture. SR responses in the SOL/TA were elicited by imposing quick ankle toes-up/toes-down rotations while standing upright and in the supine position. We designed four experimental conditions based on pre-information about perturbations: no information (No Cue), the timing of the perturbation onset (TIM), the direction of the perturbation (DIR), and both the timing and direction of the perturbation (TIM/DIR). Each condition was separated and its order was counterbalanced. In the SR of TA evoked by toes-down rotation, integrated electromyography activities of the late component were significantly reduced in the TIM and TIM/DIR conditions as compared with those in the No Cue and DIR conditions. The occurrence rate of late SR components that reflects how often the reflex response was observed was also lower in the TIM and TIM/DIR conditions as compared with that in the No Cue and DIR conditions. On the other hand, no significant changes were seen among the four conditions in the early SR component in the TA and both SR components in the SOL. The same results in the occurrence rate were found in the supine position. The present results suggest (1) only temporal predictions have a remarkable effect on the SR excitability of the TA, and (2) this effect is independent of posture.

## Introduction

Postural responses to sudden external perturbations are important for preventing falls in the course of daily living. Once standing balance has been disturbed, stretch reflexes (SR) and subsequent correcting responses are initiated approximately 40–120 ms after the perturbation onset. SRs of the ankle muscles are generally divided into three components, depending on the latencies following perturbation. The short-latency response (SLR) is regarded as a stereotypical, Ia-mediated spinal reflex altered by the magnitude of stimuli or background electromyographic (EMG) activity [1]. The later components, medium- (MLR) and long-latency responses (LLR), are known to be modified more flexibly by a subject’s preparatory states, such as prior experience [2], threat of falling [3], intention to accomplish a task [4, 5], and postural orientation [6, 7].

The predictability of the perturbation is one of the factors that influences those preparatory states. It is known that the effects of prediction on the SR of ankle flexor (dorsiflexor) and extensor (plantarflexor) muscles when standing upright differ based upon which factor the subject can predict in advance. For example, the size of the LLR in the non-stretched tibialis anterior muscle (TA) is modulated with the amplitude of perturbation (i.e., small or large perturbation) when the amplitude of perturbation is predictable, whereas it is set to respond the large perturbation when the amplitude of perturbation is unpredictable [2, 8]. On the other hand, no modulation of the SR in the stretched ankle extensor and non-stretched flexor muscle has been reported regardless of whether the direction of perturbation is predictable [9–11].

With respect to temporal prediction in the stretched muscle, few studies have investigated the influence of that on the SRs. Ackermann et al. [12] demonstrated that the latency of the SR in the stretched gastrocnemius muscle decreased with an acoustic preparatory signal. However, in their study, the direction of sudden displacement of the support surface that disturbed the standing posture was constant (i.e., from a single direction). In such a case, subjects could predict the direction of perturbation as well as the timing of the perturbation. The findings derived from this experimental design may be limited, because the spatial factor interfered with the results. Therefore, it remains unclear how the temporal prediction affects the latency and the excitability of the SR responses in the ankle muscles while standing.

The purpose of the present study was to investigate whether the SR responses of ankle flexor and extensor muscles would be modulated with temporal and/or spatial predictions of perturbation while standing. We designed experiments to distinguish clearly between temporal and spatial predictions. Rotational perturbations from two directions were randomly applied in each trial, and the timing and directional cues were presented separately. We further examined whether their effects could be seen in the supine posture. In the ankle muscles, any conclusion about the effects of predictions has been limited in the standing posture since they have been rarely reported in other postures. By comparing the effects of predictions during standing to those during supine posture, we aimed to clarify the nature of prediction-related modulation of the SR in the ankle muscles.

## Materials and Methods

### Subject and equipment

Twelve healthy subjects (25.8±3.8 years, all males) with no known neuromuscular or orthopedic disease participated in this study. All participants gave written informed consent in accordance with the Declaration of Helsinki. The protocol was approved by the Ethics Review Committee for Experimental Research with Human Subjects of the Graduate School of Arts and Sciences, The University of Tokyo.

A custom-made experimental device (Senoh Corp., Japan) that was designed to impose quick rotations of footplates aligned with axes of the ankle joints was used to evoke SRs in the ankle muscles. Footplates were connected to a servo motor to generate rotations in the toes-up (dorsiflexion) and toes-down (plantarflexion) directions. The intervals and order of footplate rotations could be set arbitrarily. In this experiment, the SR was elicited in the ankle muscles while standing quietly and in the supine position. The right and left footplates were rotated by 10 degrees at an angular velocity of approximately 200 degrees/sec. Each foot was fixed to the footplates with straps to induce the SR responses stably throughout the experiment. The torque and angular signals of the footplates were recorded at a sampling rate of 2 kHz for later analysis.

### Experiment and procedure

Four different conditions, with respect to pre-information about rotational perturbations, were conducted in this experiment as follows: no information (No Cue), a cue about the timing of the perturbation onset (TIM), a cue about the direction of the perturbation (DIR), and a cue that indicated both the timing and direction of the perturbation (TIM/DIR). The timing cue was presented with beeping sound 1.0 sec before the perturbation. The directional cue was presented with a verbal command after repositioning to quiet standing after the previous trial. Each experimental condition was carried out in a separate block with a counterbalanced order. Twenty toes-up and twenty toes-down rotations were applied with random interstimulus intervals of 10 to 20 sec in each condition, and a total of 160 perturbations were applied to both side ankle joints simultaneously. Short rests of 5 to 10 min were provided between blocks to exclude the influence of fatigue. This study was composed of two experiments as described below; these experiments were conducted on separate days.

Experiment 1 (n = 12): The effects of predictions on the SR responses of ankle muscles while standing were examined. Participants stood quietly with their arms at their sides and focused on a target that was placed at eye level on the wall 2.0 m in front of the subject. Handrails were set beside the subjects to prevent them from falling. They were instructed to keep consistent posture from trial to trial as much as possible and to try to maintain upright standing against perturbations without holding handrails unless they were likely to fall.

Experiment 2 (n = 6): To clarify whether the effect of prediction on the SR in Experiment 1 was peculiar to postural control when standing, Experiment 2 was designed to test the SR while subjects were in a supine position. The same pre-information conditions as in Experiment 1 were set in Experiment 2. Six of the 12 subjects in Experiment 1 participated in Experiment 2. They were instructed to activate their SOL muscles to an amount equivalent to the background EMG level while standing in Experiment 1 before the perturbation was applied. Target EMG level which was comparable to the EMG activity during standing was calculated before and was displayed on an oscilloscope monitor throughout all trials. The experimenter checked the EMG level and indicated to the subjects to keep the requested activation level. After the perturbation, the subjects were required to be relaxed.

### EMG activity

Surface EMG activities were recorded from the bilateral TA and SOL muscles. Standard skin preparations using alcohol and tape for abrasion were applied before the attachment of disposable electrodes (diameter: 7 mm). Bipolar Ag/AgCl surface electrodes were placed on the muscle belly with an inter-electrode distance of 10 mm. The electrodes for the TA were attached longitudinally at the one third of the distance between the tibial tuberosity and center of ankle joint and 1cm lateral edge of the tibia. Those of SOL was placed 2cm medial from the Achilles tendon and 1cm below the lower edge of medial gastrocnemius belly. Thin elastic bandages were wrapped to hold electrodes stably on the muscles and lead lines as well. EMG signals were amplified ×1000 using a bioelectric amplifier (MEG-6108, Nihon Kohden, Japan) with a bandpass filter (15–1000 Hz) and digitized at a sampling rate of 2 kHz. The signals were recorded for 500 ms before and after perturbations.

### Kinematics

In Experiment 1, kinematic data was obtained by a motion capture system (OptiTrackV100:R2, NaturalPoint, Inc., USA) in 7 subjects to ascertain whether subjects’ standing postures for the pre-perturbation period were identical in every trial. Four infrared cameras were placed at different angles on the left side of subjects. Reflective markers were attached to the acromion, the anterior superior iliac spine (ASIS), the lateral epicondyle of the femur, the middle of the lower leg, and in front of the first toe on the footplate. Because the subject’s feet were fixed to the footplates with straps, we avoided placing markers around the ankle joint. The angle displacement of the ankle joint was estimated in offline analysis by the lower leg maker, which was positioned between the medial malleolus and the knee, after careful measurement of leg length. The joint angles were calculated with four segments in a sagittal plane. The trunk segment was defined from the acromion and ASIS markers. The femoral segment was defined from the ASIS and lateral epicondyle markers. The lower leg segment was determined by using the putative lateral malleolus that was estimated on a distal projection line linked to the lateral epicondyle marker and the marker on the tibia. The distance from the lateral epicondyle marker to the putative marker was extended twice as long as that from the lateral epicondyle marker to the marker on the lower leg. The foot segment was defined between the putative lateral malleolus and the marker on the footplate. The sagittal-plane angle of the four segments was calculated as the hip, knee, and ankle joint angle. Kinematic data were recorded with EMG signals from 2 sec before to 1 sec after the perturbation with a sampling rate of 100 Hz.

### Data analysis

The digitized EMG signals were full-wave rectified after subtraction of the DC bias. The background EMG activity (BGA) and the footplate torque were calculated for 100 ms prior to the onset of the perturbation to identify the distribution of loading on the both legs from trial to trial. The ensemble-averaged EMG responses of the SOL and TA were evaluated for each condition and each rotational direction.

**Fig 1:**
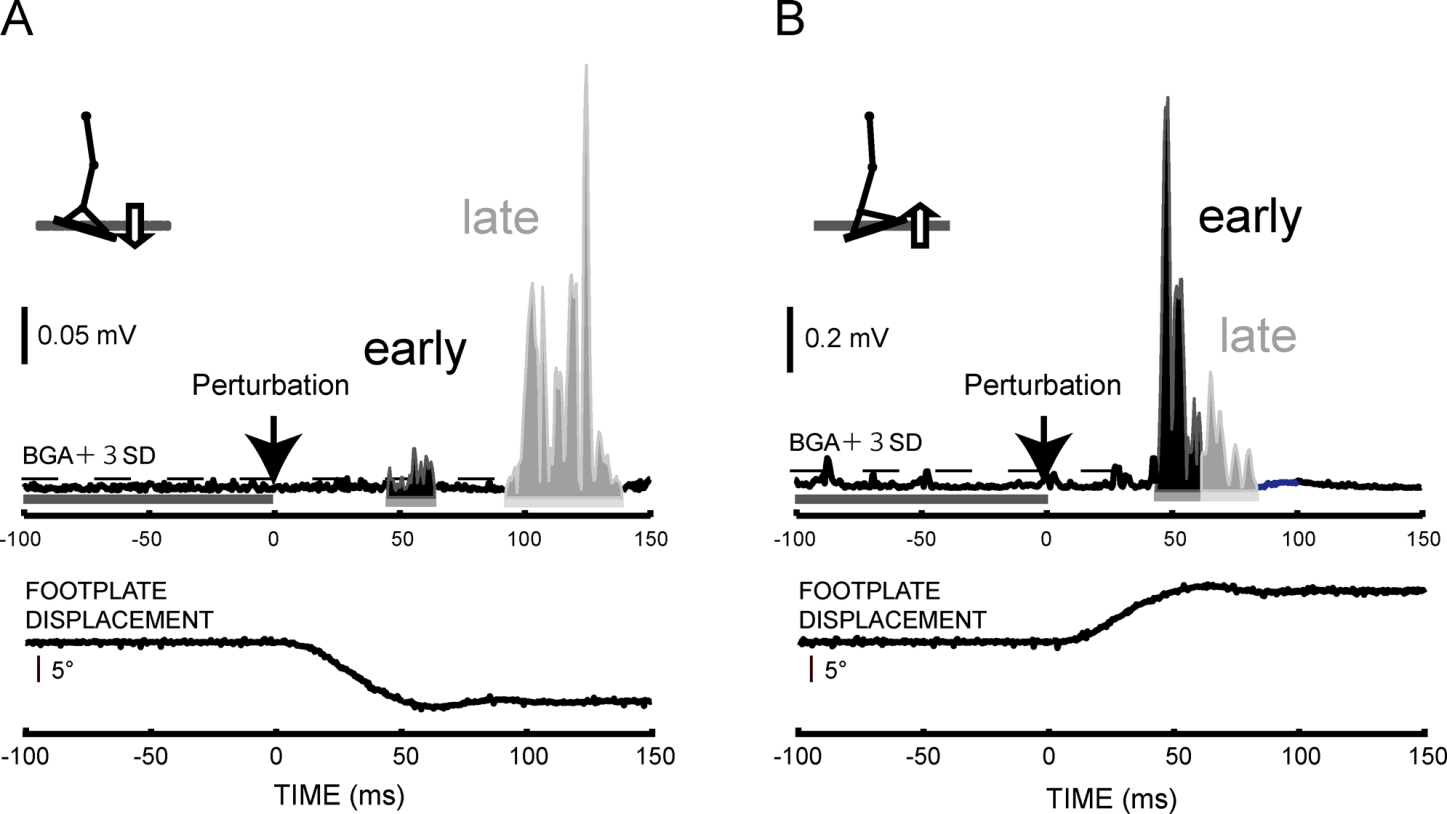
A typical example of the stretch reflex responses divided into the early and late responses. (A) In the TA responses to toes-down rotations. (B) In the SOL responses to toes-up rotations. The dashed horizontal line shows the threshold (BGA + 3SD) of each SR response. The black bar indicates the calculated period for BGA. The arrow indicates the onset of perturbation.

The present study focused on EMG responses in muscles stretched by footplate rotation—toes-up for the SOL, and toes-down for the TA. The SR responses of ankle muscles were often divided into the SLR, the MLR and the LLR in the previous studies [13, 14]. However, due to the difficulty of visual separation, we defined the two latter SR components as the late response. The early and the late responses of both muscles were determined from the ensemble-averaged EMG signals (Fig 1). The integrated EMG (iEMG) of each SR component was calculated in all trials which the occurrence of SR could be detected. The calculated period was determined from the ensemble-averaged wave in the No-Cue condition by the reference line, at which level the mean BGA plus three times its standard deviation (BGA + 3SD) was displayed on the computer monitor. In the SOL muscles, the SOL-early and the SOL-late were determined with the method adopted in the previous research [15]. The SOL-early onset was defined as the first deflection from the BGA + 3SD, and the SOL-late was visually distinguished on a display within the 20 ms after the early response onset. The iEMG was obtained by subtracting the BGA from the rectified EMG for each response in all conditions. Furthermore, the occurrence rate of each SR EMG component in each muscle was examined among all conditions. To obtain it computationally, the existence of each component was defined as having exceeded the BGA + 3SD within 40–60 ms (early response) and 60–120 ms (late response) and after the perturbation onset, respectively. We further inspected the some noise pre and post time period of the footplates movement visually. The recorded SR responses were not normalized in this study because our subjects were the almost same age and body shape and the same gender. The onset latency of two components in each muscle was determined from the ensemble-averaged wave by the BGA + 3SD for every experimental condition. In Experiment 1, the trials in which the subjects activated the TA or the SOL before applying perturbation above the mean BGA + 3SD were discarded (toes-up: 0.7%, toes-down: 1.8% of all trials).

Kinematic data obtained from the motion capture system and footplates was used to determine the joint angles of lower limbs, footplate angles, and velocities. The mean angles of hips, knees, and ankle joints as defined above were compared in each condition over 300 ms before the perturbations. The mean footplate velocities during the 50 ms after the onset were calculated to verify that the same magnitude of perturbation was applied in every trial.

### Statistical analysis

The effects of the cue condition on the iEMG and the latency of each SR component were tested using a one-way analysis of variance (ANOVA) with repeated measure. A one-way ANOVA was also performed to test the effect of cue conditions on the BGA, joint angle, angular displacement, and footplate velocity. When statistical significance was confirmed, Bonferroni corrected post hoc comparisons were performed to identify the differences between different conditions. A chi-square test was applied to compare the occurrence rates of the SR responses. The significance level was set at p<0.05. Results were presented as means ± SE.

## Results

### Effect of temporal and spatial prediction on SR responses while standing

The SR of the TA elicited by toes-down rotation and that of the SOL elicited by toes-up rotation were analyzed. Because there were no detectable differences between EMG responses recorded of legs on both sides and the kinematic data was recorded from the left side, only the result of the left side was shown in this study. The BGAs of both muscles while standing were not significantly different among all conditions (TA: F_3 33_ = 2.26, p = 0.10, SOL: F_0.1 1.4_ = 14.9, p = 0.83). There was no significant effect in the footplate displacements and the velocities among all conditions. The mean footplate torque for 100ms before perturbation was also not significantly different between the left and right sides, which meant the symmetrical load to both legs. Furthermore, the joint angles of the lower limbs preceding onset of the mechanical perturbation showed no significant differences among all conditions. Therefore, the analyzed data through post process can be postulated to be induced by the almost same perturbation.

**Fig 2:**
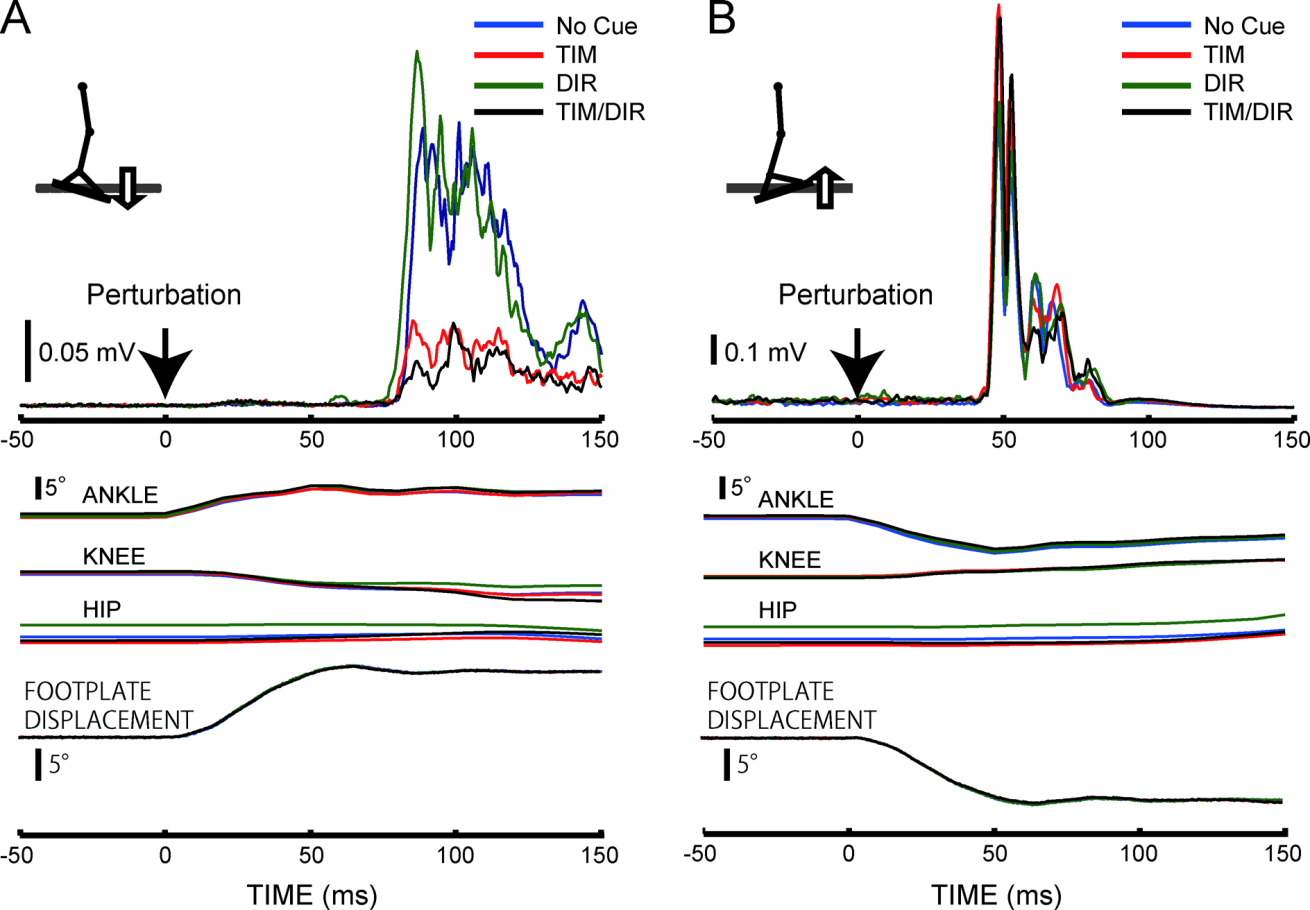
Representative ensemble-averaged waveforms of the stretch reflex responses with movement trajectories of the ankle, knee, and hip joints under the different cue conditions in a single subject. (A) In the TA muscles due to toes-down rotations. (B) In the SOL muscle due to toes-up rotations.

**Fig 3:**
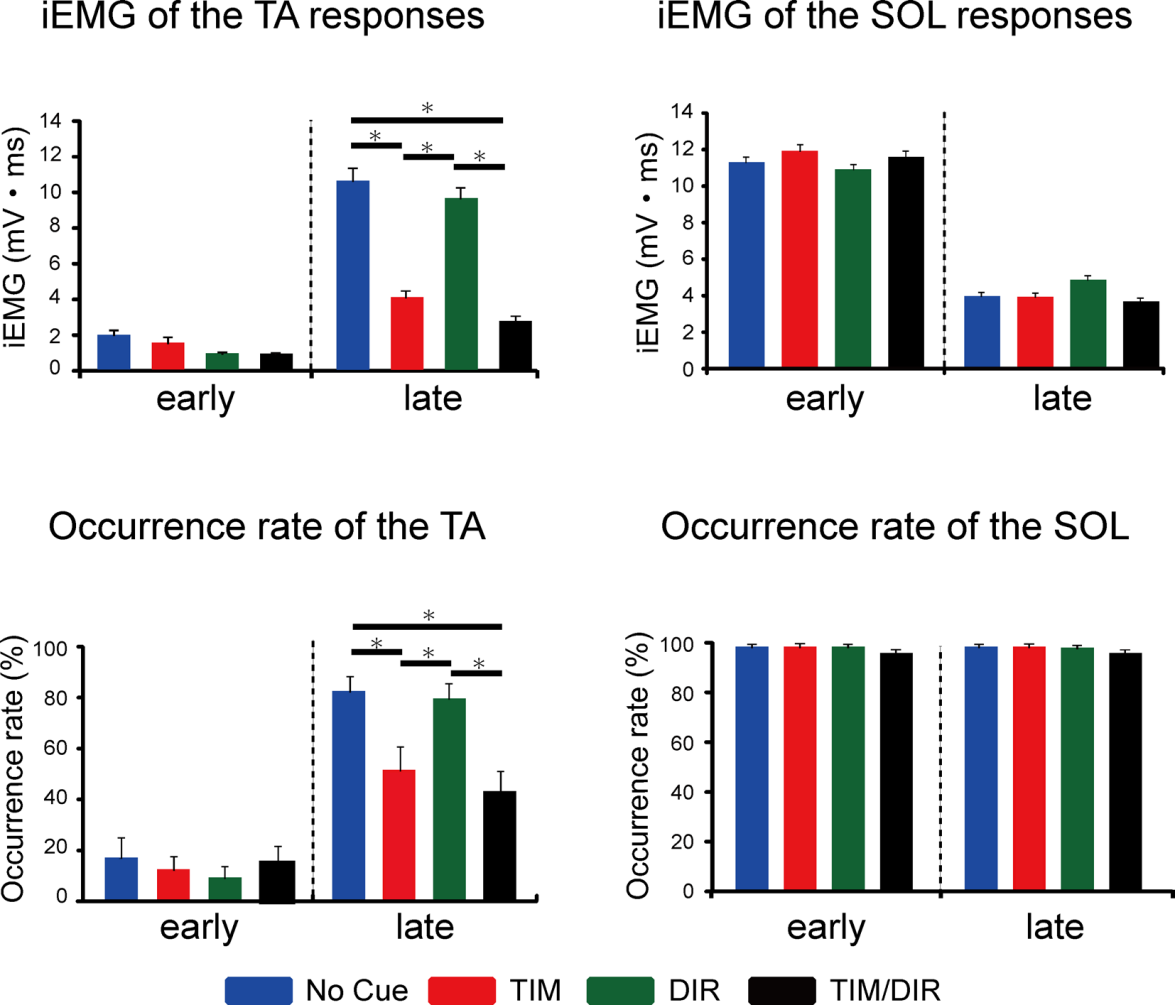
Comparisons of the iEMG and occurrence rate of each reflex component in the TA and SOL among the different cue conditions while standing. The iEMG error bars indicate the SE of the mean value. The occurrence rate error bars indicate the SE of the mean value. *Statistically significant difference (p<0.05)

Fig 2 shows representative ensemble-averaged EMG and kinematic recordings among different conditions from a single subject (Fig 2). It was demonstrated that the TA-late in the No-Cue and DIR conditions were larger than in the TIM and TIM/DIR conditions. In contrast to the TA-late, there were no noticeable changes in the SOL-late across all conditions. The iEMG of the TA-late revealed a significant main effect of the conditions (F_1.4 15.4_ = 14.7, p<0.05) (Fig 3). A post hoc test showed statistical reduction of SR amplitude in the TIM and TIM/DIR conditions as compared with the No-Cue condition (p<0.05). Both the TIM and TIM/DIR conditions also brought significantly smaller responses than those in the DIR condition (p<0.05), whereas no considerable difference was observed between the TIM and TIM/DIR conditions. Additionally, no significant difference between the No-Cue and DIR conditions was found. It was suggested that the spatial cue did not contribute to modulating the TA-late, and the combination effect of spatial and temporal prediction could not be verified. In contrast to the TA-late, the iEMG of the TA-early was not significantly different among all experimental conditions. On the other hand, both the SOL-early and SOL-late were not different among the conditions. The average latencies of both muscles were similar to those reported in previous studies [13, 16]. Neither component of TA or SOL muscles significantly changed among all conditions (Table 1).

**Table 1:** Latencies of early and late responses while standing and in the supine position among the different cue conditions.

A					
Position	component	condition			
		No Cue	TIM	DIR	TIM/DIR
Standing (n = 12)	early [ms]	45.4±1.4	49.2±1.6	47.7±1.2	46.5±1.0
	late [ms]	86.6±1.4	89.5±2.4	87.3±1.6	92.0±3.4
Supine (n = 6)	early [ms]	45.6	44.8	44.5	46.4
	late [ms]	85.4±2.7	86.6±3.0	86.0±3.1	93.8±0.4
B					
Position	component	condition			
		No Cue	TIM	DIR	TIM/DIR
Standing (n = 12)	early [ms]	43.2±0.6	42.7±0.6	43.2±0.6	43.3±0.7
	late [ms]	60.3±0.8	60.0±0.8	60.3±0.7	60.5±0.6
Supine (n = 6)	early [ms]	43.3±0.9	43.5±0.8	43.0±0.8	43.6±0.9
	late [ms]	59.9±0.9	59.0±0.9	59.5±0.9	59.4±1.0

A; In the TA muscle due to toes-down rotations. B; In the SOL muscle due to toes-up rotations.

### Effects of temporal and spatial predictions on the occurrence rate of SR responses while standing

Consistent with the previous study [17], the occurrence rate of the TA response elicitation was different from that of the SOL response elicitation. In Experiment 1, the TA-early responses were abolished in three of twelve subjects (No Cue: 23.8%, TIM: 22.5%, DIR: 20.9%, TIM/DIR: 29.1%). As to the effect of prior knowledge of perturbation in the TA-early, significant differences were not seen among the different conditions. The TA-late responses were elicited in all subjects, while the occurrence rate was markedly different among the conditions (No Cue: 85.5%, TIM: 51.3%, DIR: 81.7%, TIM/DIR: 41.8%). Those in all but two subjects were significantly reduced in the TIM and TIM/DIR conditions (Fig 3) (χ^2^ = 115.94, p<0.05). Thus, it seemed that the occurrence rate of the TA-late elicitation was predominantly affected by the temporal prediction. The occurrence rate of the SOL-early and SOL-late elicitations was not affected by either temporal or spatial prediction (No Cue: 96.7%, TIM: 97.1%, DIR: 96.3%, TIM/DIR: 94.6%, the same probability in the early and the late responses).

### Difference in the effect of predictions on SR responses while standing or in the supine position

**Fig 4:**
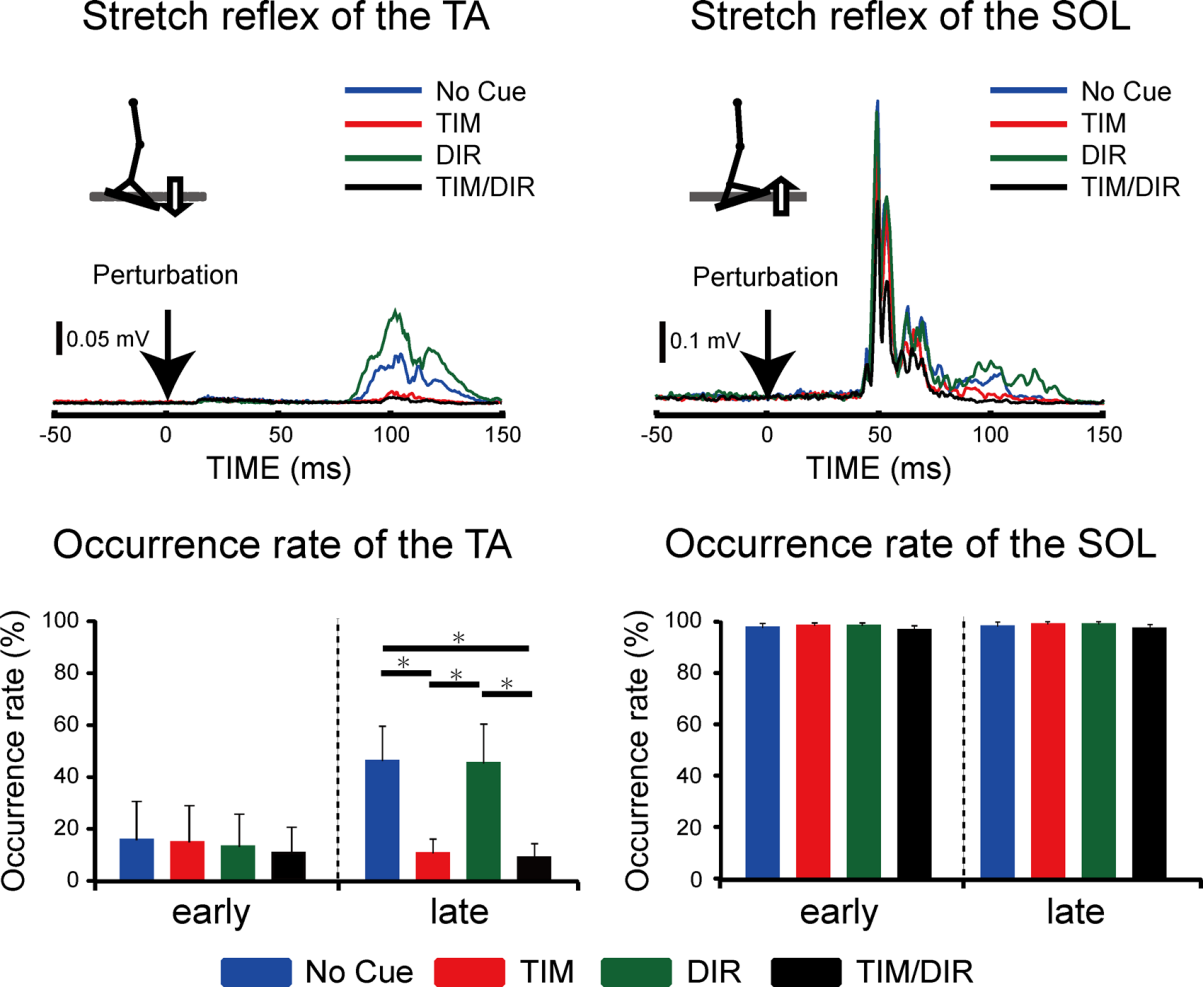
Representative ensemble-averaged waveforms of the stretch reflex responses in a single subject, and comparisons of the iEMG and occurrence rate of each reflex component in the TA and SOL among the different cue conditions in the supine position. The iEMG error bars indicate the SE of the mean value. The occurrence rate error bars indicate the SE of the mean value. *Statistically significant difference (p<0.05)

For the purpose of examining whether the result of Experiment 1 was specific to the standing posture, a second experiment was conducted to test the SR in a supine position under the same cue conditions as in Experiment 1. Fig 4 illustrates a typical EMG trace in each condition from a representative subject (Fig 4). The previous study showed that both the size and occurrence rate of SR of TA were dramatically decreased in the supine position as compared to those when standing [17]. Actually, we found that the occurrence rate of TA-late elicitation was clearly reduced in the supine position as compared to that while standing in all conditions (No Cue: 45.8%, TIM: 10.0%, DIR: 45.0%, TIM/DIR: 8.3%). In the TIM and TIM/DIR conditions, the occurrence rates of TA-late were significantly decreased as compared to those in the No-Cue and DIR conditions (χ^2^ = 96.76, p<0.05). In three out of six subjects, neither the TA-early nor the TA-late was observed in the TIM and TIM/DIR conditions. For the other three subjects, the sizes of the TA-late in the TIM and TIM/DIR conditions were remarkably reduced as compared with those in the No Cue and DIR conditions (No Cue: 2.9±0.7 mV·ms, TIM: 1.8±0.3 mV·ms, DIR: 2.8±0.6 mV·ms, TIM/DIR: 0.7±0.2 mV·ms). However, we did not perform statistical analysis to compare the sizes across conditions because of the low rate of occurrence of the TA response. As a whole, Experiments 1 and 2 showed similar results regarding the effect of predictions on the TA-late: it was reduced when the subject knew the timing of the perturbation onset. The TA-early was not observed in the supine position, except in one subject (No Cue: 2.5%, TIM: 2.5%, DIR: 0.0%, TIM/DIR: 0.8%). The iEMG and occurrence rate of both SOL-early and SOL-late were not affected by any conditions, which was the same as in Experiment 1. The BGAs in the supine position were comparable across all conditions. The average onset latencies were equivalent among each condition in the supine position in the early and late responses of both muscles (Table 1).

## Discussion

The aim of the present study was to investigate the effect of predicting a rotational perturbation on the stretch reflex in the ankle muscles. We focused on how the temporal and spatial predictions, separately and combined, affected the SOL and TA SR responses while standing upright and whether their effects were specific to the standing posture. The results showed that, in the TA-late, the iEMG was smaller and the occurrence rate was lower in the TIM and TIM/DIR conditions as compared to the No-Cue or DIR conditions while standing. The lower occurrence rate of the TA-late in the TIM and TIM/DIR conditions was also found in supine posture. On the other hand, neither temporal nor spatial prediction affected the TA-early, SOL-early, and SOL-late. These modulations of reflex responses were not associated with variations in the EMG levels and/or kinematics before a rotational perturbation because they were kept constant among the different conditions in the standing and supine postures. It is unknown whether the present results have been affected by habituation which means the gradual decrease in SR amplitude with the same repeated stimulus [18, 19, 20]. However, we suppose that the habituation phenomenon on the reduction of the TA-late would be negligible because the opposite directions of the perturbations have been equally randomized in all conditions.

These results suggest the following three features effect the spatial and temporal predictions on the SR responses in the ankle muscles: (1) temporal predictions are crucial for modulating SR responses; (2) the effect is different between the SOL and the TA; and (3) the effect is independent of posture. The possible neurophysiological mechanisms underlying these results are discussed in the following sections.

### Different effects of the temporal prediction on the SR responses of the SOL or TA muscles

The present results showed a remarkable reduction of the iEMG and occurrence rate in the TA-late if the subject could predict the timing of a rotational perturbation, while it was not found in the TA-early, SOL-early, or SOL-late. Our results were in line with previous observations that the TA responses were strongly influenced by changes in the preparatory state, while the SOL responses were not [16, 21]. Nardone et al. [16] reported that longer latency reflex responses, evoked by rotational and translational displacement of a platform while the subjects stood quietly, were reduced when the subjects held the stable frame. Nakazawa et al. [21] demonstrated that the TA EMG responses induced by sudden drops of support surface were reduced in the condition with a warning cue before perturbation or when touching a bar.

These differences between the SOL and TA may be attributed to the different neurophysiological features behind the control of these muscles. It is well recognized that the activity of the TA is more under the control of the supraspinal mechanism, and that the SOL response is dominantly related to spinal mechanisms modulated by peripheral information [22, 23]. The corticomotoneuronal connection seems to be stronger in the TA than in the SOL muscle. It has been reported that higher stimulus intensity or larger excitability in spinal motoneurons is needed to elicit the SOL response by transcranial magnetic stimulation of the motor cortex, while the excitatory TA response could be prominently elicited in a way similar to that in the upper limb muscles [24–26].

Different reflex pathways are other candidates. The TA-late observed in the present study is probably, at least in part, mediated through the transcortical reflex pathway. The presence of this pathway in the longer latency responses, more than 80 ms after perturbation, was strongly suggested by several researchers [14, 27–29]. The onsets of the SOL-early and the TA-early were 43.2 ms and 45.4 ms, respectively, and are believed as an Ia-mediated spinal reflex. The onset of the SOL-late was 60.3 ms, which is short in comparison to the onset of the transcortical reflex pathway that was reported in a previous study [30]. Our findings that the temporal prediction reduces the SR of TA but not that of SOL may be depending on the different strengths of the corticospinal connection and the reflex pathway. The TA-late is thought to be sensitive to changes in the supraspinal neural activities, which would be modulated depending on whether the subject can predict the timing of the perturbation.

### Effect of the temporal prediction on the TA-late response

In a great majority of studies, the effect of the predictability of a perturbation has been examined in the upper limb muscles, and they have reported that a long-latency reflex response is modified according to the instruction given to the subjects (e.g., [31, 32]), whereas a short-latency reflex response is unchanged. In the few studies that deal with the ankle muscles, the modulation of late-component TA and SOL responses with temporal prediction has been reported only in the standing posture [12, 21]. A new finding in the present study was that, when the subjects could predict the timing of a perturbation, the excitability of the TA-late was modulated not only in the standing posture but also in the supine posture. These results suggest similar cortical involvement in both standing and supine postures in terms of the predictability of a perturbation.

Functionally, decreases in the iEMG and the occurrence rate of the TA-late seem to be appropriate for executing required tasks in the present study. The SR responses evoked by rotational perturbation in the ankle muscles destabilize standing posture rather than recovering from the disequilibrium [33, 34]. In the supine posture, subjects were asked not to resist a mechanical perturbation (i.e., let go). In both cases, the reduced TA-late would help to accomplish the required task. When the timing of perturbation onset was not unexpected, the larger TA amplitude might be prepared so that the ankle joint could be stabilized anytime, similar to the results in a report by Beckley et al. [8] that the larger TA response was set in advance to be on the safe side when the perturbation size was unexpected. Our results suggest that the central nervous system can modulate the reflex activity of stretched muscles, allowing the required task to be executed efficiently, when information about the timing of a perturbation is available.

One possible interpretation of our results is that the attenuation of startle-like reflexes superimposed on the SR resulted in a decrease in the TA-late response. It has been suggested that psychological factors, such as a fear of falling and being startled at a sudden surface displacement, increased the reflex bursts while standing or walking [3, 18, 35]. If the temporal uncertainties of perturbation onset give rise to feeling threatened or startled, it is reasonable that a part of the TA-late as threat- and/or startle-induced reflex bursts could be reduced with temporal prediction both in the standing and supine postures.

Several studies reported that the early part of TA-late around 80–90 ms after the perturbation is likely to be considered as the type II -related EMG response [36, 37]. Since the most part of TA-late above 90 ms is thought to be transcortical, it is plausible that not only the cortical circuit but also the spinal neuronal network contribute to elicitation of the TA-late. According to the previous reports using transcranial magnetic stimulus, the later component of the SR through the corticospinal pathway was clearly suppressed by activation of inhibitory systems at the cortical level [14, 29]. We postulated the spinal inhibitory system also works to the reduced TA-late. Corna et al. reported that the SR component relayed by the type II afferent could be influenced by inhibitory effects of the postural set, while the Ia-mediated spinal reflex is not affected [37]. As the same way, the temporal prediction might lead a different gain in a spinal neuronal network involving the type II afferents.

### Effect of spatial prediction on SR responses

In contrast to temporal prediction, spatial prediction about perturbation direction had no apparent effect on SRs in the ankle muscles in either the standing or supine position. Even when the temporal and spatial predictions were combined, SRs did not differ significantly from those with the temporal prediction alone. The present results are consistent with those of Diener et al. [9], who reported no statistically significant differences in the latencies and iEMGs with or without advanced visual information about the direction of the upcoming footplate rotation. Because the sensory inputs that depend on the perturbation direction trigger muscle activation patterns for postural responses [38–40], we hypothesized that the spatial prediction helped to speed the process of evoking the SR. However, it was convincing that the spatial prediction had no effect on SR responses if the direction-specific muscle activation patterns were determined in an automatic manner without the higher nervous center. Evidence that similar directional tuning curves of muscle activities evoked by surface translation were seen between decerebrate and intact cats supports this idea [41, 42].

In addition, our results might have stemmed from the instructions given to the subjects. The subjects were asked to remain in the standing posture and to be passive relative to perturbation in the supine posture. In these situations, advance information regarding the perturbation direction may be irrelevant because the task goals can be settled regardless. If the postural adjustment before perturbation was controlled by the subjects, the spacial prediction might contribute to the modulation of the SR. Because our results are limited in the SR component of distal leg muscles within 120ms excitation after the perturbation, it may be possible that the following EMG components after the SR and/or the more proximal muscles are affected with spatial prediction.

## Limitation

The interpretations of our findings include some limitations. First, the number of subjects in Experiment 2 may be few for statistical analysis. This is because that the experimental device for applying footplate displacement had broken down on the way to complete all subjects. In the half of the subjects participated in Experiment 1, the TA-late was similarly modulated to that in Experiment 1. We could observe that the occurrence rate, at least, of the TA-late was significantly reduced with temporal prediction in supine position. However, it is unknown that in the others the TA-late would be reduced in the same way. Second, in this study, we focused only on the SRs of the TA and the SOL which are monoarticular, antagonist muscles. Our results could not be extended to other ankle extensor and flexor muscles. Another ankle extensor, such as the medial gastrocnemious muscle which is biarticular, fast fiber-dominated, might be modulated differently with temporal and spatial predictions.

## Conclusions

The results of the present study demonstrate that the temporal prediction of an upcoming perturbation reduced the iEMG and the occurrence rate of the TA-late while both standing and supine, regardless of whether subjects could predict its direction. Our findings suggest that TA muscle is susceptible to anticipation of the postural instability and temporal certainty is key factor for SR modulation independent of posture.

## Supporting Information

S1 Fig Comparisons of the footplate torque among the different conditions while standing. (TIF)

S2 Fig Comparisons of the Background EMG activities in the TA and SOL muscles among the different conditions while standing and in the supine posture. (TIF)

S3 Fig Comparisons of the iEMG and occurrence rate of each reflex component in the opposite side of the TA and SOL among the different cue conditions while standing. (TIF)

S1 Table Comparisons of the joint angles of lower limb among the different conditions while standing. (DOCX)

S2 Table Comparisons of the footplate displacements and velocities among the different conditions. (DOCX)
