# Supplementary figures and images for: The Effects of Temporal and Spatial Predictions on Stretch Reflexes of Ankle Flexor and Extensor Muscles While Standing
Source: PLoS One. 2016 Jul 6;11(7):e0158721. doi: 10.1371/journal.pone.0158721 (PMC4934788; doi:10.1371/journal.pone.0158721)

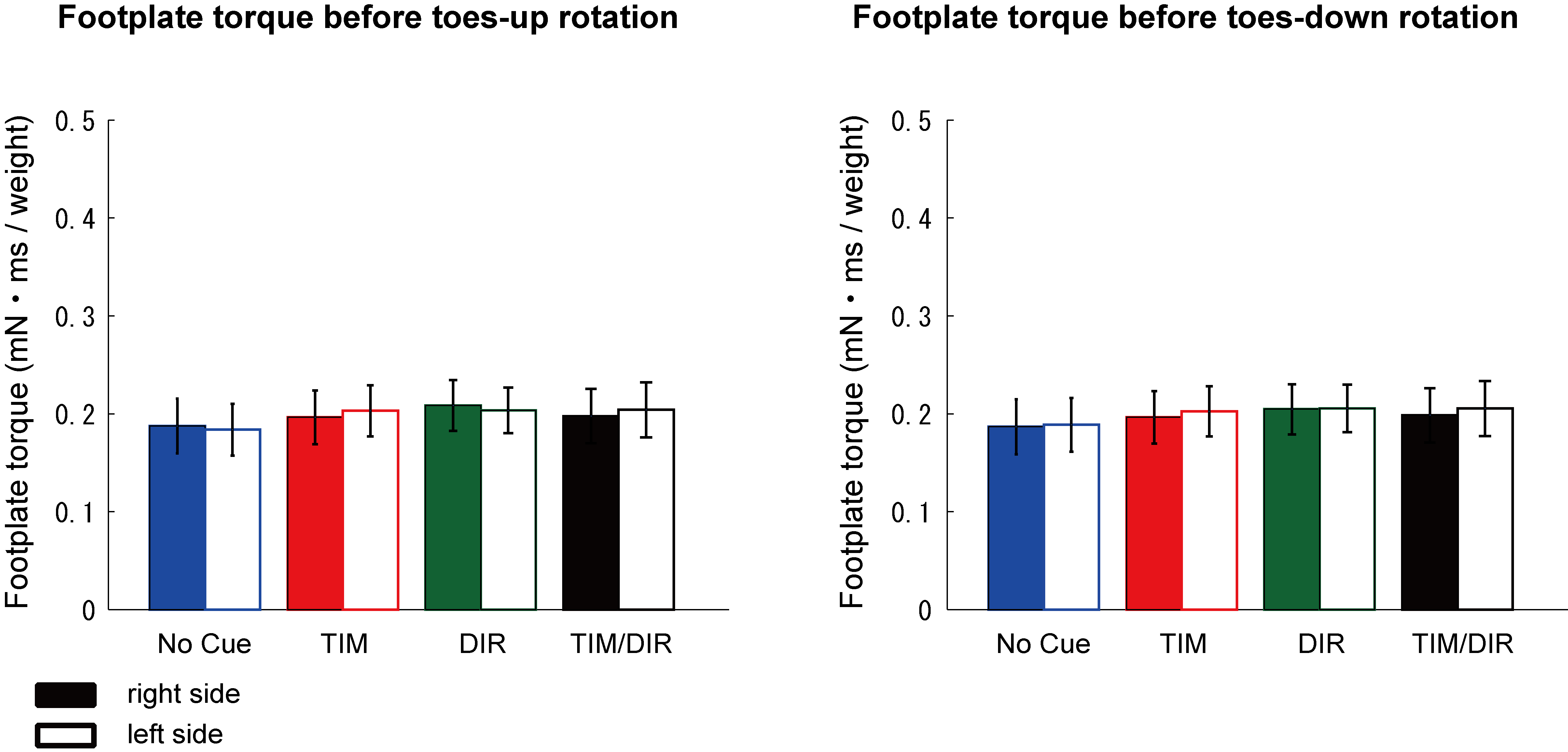

Supplement: S1 Fig — (TIF) [file pone.0158721.s001.tif]

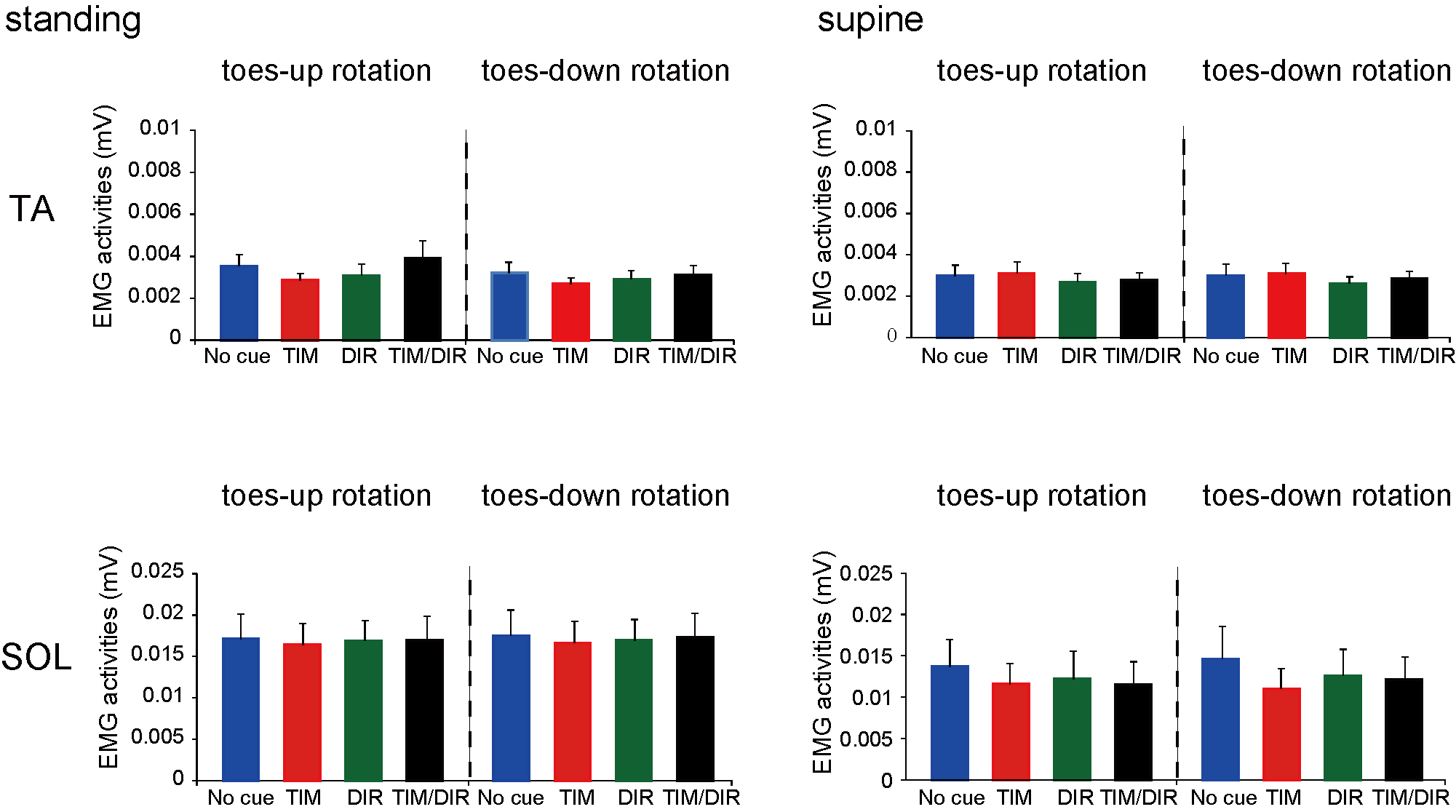

Supplement: S2 Fig — (TIF) [file pone.0158721.s002.tif]

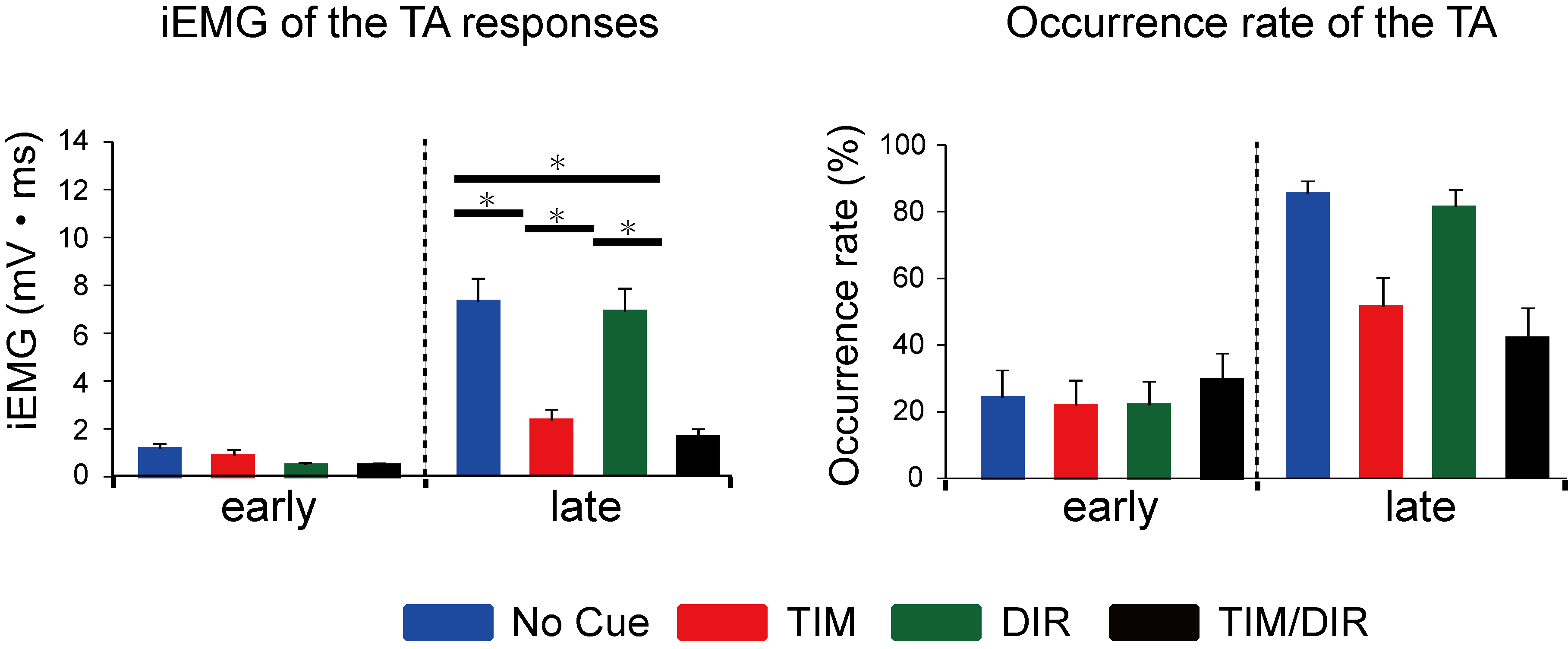

Supplement: S3 Fig — (TIF) [file pone.0158721.s003.tif]
