# Supplementary material for: The Effects of Temporal and Spatial Predictions on Stretch Reflexes of Ankle Flexor and Extensor Muscles While Standing
Source: PLoS One. 2016 Jul 6;11(7):e0158721. doi: 10.1371/journal.pone.0158721 (PMC4934788; doi:10.1371/journal.pone.0158721)
Supplement: S1 Table — (DOCX) [file pone.0158721.s004.docx]

S1 Table. Comparisons of the joint angles of lower limb among the different conditions while standing.

Joint angles of lower limb in toes-down rotations (°)

|  | No Cue | TIM | DIR | TIM/DIR | p-value |
| --- | --- | --- | --- | --- | --- |
| Ankle | 78.5±0.5 | 78.1±0.5 | 78.8±0.5 | 78.5±0.6 | 0.77 |
| Knee | 174.5±0.4 | 174.7±0.4 | 174.6±0.4 | 175.0±0.5 | 0.67 |
| Hip | 157.9±0.3 | 156.3±0.4 | 156.8±0.5 | 156.4±0.4 | 0.15 |

Joint angles of lower limb in toes-up rotations (°)

|  | No Cue | TIM | DIR | TIM/DIR | p-value |
| --- | --- | --- | --- | --- | --- |
| Ankle | 79.0±0.5 | 78.5±0.5 | 78.7±0.5 | 78.5±0.5 | 0.83 |
| Knee | 174.8±0.4 | 174.7±0.5 | 174.4±0.4 | 174.9±0.4 | 0.34 |
| Hip | 157.7±0.4 | 156.3±0.4 | 157.1±0.5 | 156.8±0.4 | 0.10 |
