# Supplementary material for: The Effects of Temporal and Spatial Predictions on Stretch Reflexes of Ankle Flexor and Extensor Muscles While Standing
Source: PLoS One. 2016 Jul 6;11(7):e0158721. doi: 10.1371/journal.pone.0158721 (PMC4934788; doi:10.1371/journal.pone.0158721)
Supplement: S2 Table — (DOCX) [file pone.0158721.s005.docx]

S2 Table. Comparisons of the footplate displacements and velocities among the different conditions.

Footplate displacements and velocities to toes-up and toes-down while standing; Displacement (°), Velocity (°/sec)

|  |  | No Cue | TIM | DIR | TIM/DIR | p-value |
| --- | --- | --- | --- | --- | --- | --- |
| Toes-up | displacement | 9.7±0.03 | 9.7±0.02 | 9.7±0.03 | 9.7±0.04 | 0.17 |
|  | velocity | 231.6±1.94 | 230.9±1.58 | 229.1±1.48 | 229.9±1.54 | 0.07 |
| Toes-down | displacement | 11.1±0.03 | 11.2±0.03 | 11.1±0.05 | 11.2±0.05 | 0.07 |
|  | velocity | 264.5±1.82 | 263.9±1.60 | 262.9±1.43 | 264.0±1.28 | 0.57 |

Footplate displacements and velocities to toes-up and toes-down while supine position; Displacement (°), Velocity (°/sec)

|  |  | No Cue | TIM | DIR | TIM/DIR | p-value |
| --- | --- | --- | --- | --- | --- | --- |
| Toes-up | Displacement | 9.6±0.18 | 9.8±0.10 | 9.7±0.11 | 9.5±0.22 | 0.18 |
|  | Velocity | 231.0±3.94 | 236.1±1.99. | 236.1±1.99 | 228.5±5.30 | 0.20 |
| Toes-down | Displacement | 11.2±0.19 | 11.4±0.17 | 11.4±0.07 | 11.4±0.09 | 0.11 |
|  | Velocity | 272.3±4.88 | 277.7±1.81 | 278.5±2.12 | 277.9±2.39 | 0.13 |
